# The Effect of a Novel c.820C>T (Arg274Trp) Mutation in the Mitofusin 2 Gene on Fibroblast Metabolism and Clinical Manifestation in a Patient

**DOI:** 10.1371/journal.pone.0169999

**Published:** 2017-01-11

**Authors:** Małgorzata Beręsewicz, Anna Boratyńska-Jasińska, Łukasz Charzewski, Maria Kawalec, Dagmara Kabzińska, Andrzej Kochański, Krystiana A. Krzyśko, Barbara Zabłocka

**Affiliations:** 1 Molecular Biology Unit, Mossakowski Medical Research Centre, PAS, Warsaw, Poland; 2 Department of Physics, University of Warsaw, Warsaw, Poland; 3 Neuromuscular Unit, Mossakowski Medical Research Centre, PAS, Warsaw, Poland; University of Valencia, SPAIN

## Abstract

Charcot-Marie-Tooth disease type 2A (CMT2A) is an autosomal dominant axonal peripheral neuropathy caused by mutations in the mitofusin 2 gene (*MFN2*). Mitofusin 2 is a GTPase protein present in the outer mitochondrial membrane and responsible for regulation of mitochondrial network architecture via the fusion of mitochondria. As that fusion process is known to be strongly dependent on the GTPase activity of mitofusin 2, it is postulated that the MFN2 mutation within the GTPase domain may lead to impaired GTPase activity, and in turn to mitochondrial dysfunction. The work described here has therefore sought to verify the effects of MFN2 mutation within its GTPase domain on mitochondrial and endoplasmic reticulum morphology, as well as the mtDNA content in a cultured primary fibroblast obtained from a CMT2A patient harboring a *de novo* Arg274Trp mutation. In fact, all the parameters studied were affected significantly by the presence of the mutant MFN2 protein. However, using the stable model for mitofusin 2 obtained by us, we were next able to determine that the Arg274Trp mutation does not impact directly upon GTP binding. Such results were also confirmed for GTP-hydrolysis activity of MFN2 protein in patient fibroblast. We therefore suggest that the biological malfunctions observable with the disease are not consequences of impaired GTPase activity, but rather reflect an impaired contribution of the GTPase domain to other MFN2 activities involving that region, for example protein-protein interactions.

## Introduction

Charcot-Marie-Tooth disease type 2A (CMT2A) is an autosomal dominant axonal peripheral neuropathy characterized by axonal degeneration of sensory and motor nerves. The disease is further characterized by a wide range of symptoms, which vary with severity and time of onset [1,2]. CMT2A is caused by mutations in the mitofusin 2 (*MFN2*) gene encoding mitofusin 2 (MFN2). The mechanism underpinning the pathogenicity of *MFN2* gene mutations is not clear, but is certainly associated with impaired function of the MFN2 protein.

MFN2 is large nuclear-encoded dynamin-like GTPase anchored in the outer mitochondrial membrane. It is involved mainly in mitochondrial fusion, and the tethering of the endoplasmic reticulum to mitochondria. Data indicate the participation of mitofusin 2 in the maintenance of mtDNA integrity and regulation of respiratory chain activity.

So far, about 100 mutations of the *MFN2* gene have been reported in CMT2A patients [3]. Most of these located in and around the GTPase domain of mitofusin 2. Interestingly, there is no clear correlation between disease severity and location of the mutation within the gene.

The GTPase domain of mitofusin 2 is a highly-conserved structure responsible for the binding and hydrolysis of GTP. GTPase activity is thus suggested as a central player in the mechanism by which mitofusin 2 gives rise to mitochondrial fusion [4]. Mutations designed to block GTP nucleotide binding or hydrolysis have been shown to disrupt mitochondrial fusion [5–7], though there are few data to indicate how GTPase activity contributes to the fusion reaction.

Recently, we detected a novel mutation within the GTPase domain of *MFN2*—p.Arg274Trp (c.820C>T)–in a patient with early-onset CMT2A in which peripheral axonal neuropathy coexists with mild mental retardation [1]. While it emerged that a mutation in the same codon had been reported by Züchner (p.Arg274Gln (c.821G>A)) [8], the clinical course noted in the case of Züchner’s patient was clearly milder than that found in our patient. Questions therefore arise as to how these two mutations affect mitofusin 2 function, and evoke such different clinical outcomes.

We presumed that, because the two mutations are within the GTPase domain, they may affect the GTPase activity of mitofusin 2. To address such issues, we first performed whole-genome sequencing of the patient harboring the p.Arg274Trp mutation, in order to preclude the presence of mutations other than c.820C>T (p.Arg274Trp) *MFN2* capable of affecting the clinical course of the disease and giving rise to mild mental retardation. A cultured primary fibroblast obtained from the same CMT2A patient was then characterized in terms of parameters on which the GTPase activity of mitofusin 2 could have a significant impact. We found that mitochondrial and endoplasmic reticulum morphology and mtDNA content were affected significantly by the presence of the mutant MFN2 protein. Subsequently, using the stable model of mitofusin 2 obtained by us, we revealed that neither the mutation identified by us nor that studied by Züchner [8] has a direct impact on GTP binding. We therefore suggest that the biological malfunctions observed are not consequences of impaired GTPase activity, but rather reflect an impaired contribution from the GTPase domain in other MFN2 activities involving that region, for example protein-protein interactions.

## Materials and Methods

### Whole exome sequencing

Whole exome sequencing (WES) of the proband’s DNA was performed in line with the protocol from Illumina’s *TruSeq Exome Enrichment Guide*. A SureSelect Human All Exon 50 Mb Kit (from Agilent Technologies). Hybridized fragments were bound to streptavidin beads, while non-hybridized fragments were washed out. The library was then verified on an Agilent 2100 Bioanalyzer, and sequenced on a HiSeq 2000 instrument (Illumina) from Intelliseq LLC (Kraków, Poland).

### Exome sequence data analysis

The sequence reads were analysed using the routine Illumina pipeline. Reads were aligned to a human reference sequence (GRCh37), and processed using standard softwares (Bowtie2, Picard, SAMtools, GATK) [9–11]. The data obtained were analyzed using the Galaxy platform [12–14], by annotation with multiple INFO fields required for filtering (dbSNP allele frequencies (http://www.ncbi.nlm.nih.gov/projects/SNP/), 1000 Genomes allele frequencies (www.1000genomes.org), Exome Aggregation Consortium (ExAC) allele frequencies (http://exac.broadinstitute.org), Mitomap allele frequencies (http://www.mitomap.org/MITOMAP), the OMIM database (from dbSNP 142) and the public HGMD database (http://www.hgmd.cf.ac.uk/ac/index.php), using the SnpSift tool. Variant effect prediction was then performed using Genetic variant annotation and the effect prediction toolbox (SnpEff) [15].

In the next step, 113 genes associated with different types of hereditary neuropathies were analyzed, including those investigated by Rudnik-Schöneborn *et al*. 2015 [16]. Moreover, 20 mitochondrial genes and *POLG* (DNA polymerase subunit gamma-1) were screened with similar capture as nuclear genes for sequence variants that could influence phenotype in an additive manner. In line base data were filtered to remove common (frequency >0.01) polymorphisms by screening against dbSNP (http://www.ncbi.nlm.nih.gov/projects/SNP/); 1000 Genomes (www.1000genomes.org) and the ExAC databases; and data from 27 local control exomes.

#### *In silico* analysis

To predict possible pathogenic roles, selected variants identified in WES and associated with human disorders were analyzed using the following bioinformatics tools: PANTHER (http://www.pantherdb.org/tools/csnpScoreForm.jsp), PolyPhen 2 (http://genetics.bwh.harvard.edu/pph2/), SIFT (http://sift.bii.a-star.edu.sg/www/SIFT_seq_submit2.html) and Mutation Assessor, release 2 http://mutationassessor.org, the Mutation Taster http://www.mutationtaster.org/.

### Skin fibroblast cultures

Skin fibroblasts were derived, following informed consent, from three healthy donors (controls) and one patient harboring the p.Arg274Trp (c.820C>T) mutation in the *MFN2* gene. All procedures received prior approval from the Cardinal Stefan Wyszynski University in Warsaw Local Ethical Committee (3/2012 CSWUW, valid till 2017). Primary fibroblast cultures were established using the explant method in high-glucose DMEM (Life Technologies) supplemented with 20% fetal bovine serum (FBS, Life Technologies), glutamax, antibiotic-antimycotic (Life Technologies) and 50 μg/ml uridine (Sigma). Following satisfactory proliferation, cells were cultured in DMEM, supplemented with 10% FBS (Life Technologies) and uridine at 50 μg/ml, at 37°C and in a 5% CO_2_ humidified atmosphere. For the purposes of experiments, fibroblasts were grown either in regular medium (DMEM, 10% FBS) or in nutrient-deficient medium (DMEM glucose-free containing 5mM galactose (Sigma), 5mM pyruvate (Life Technologies) and 2% FBS), hereinafter referred to as the glucose and glucose-free media respectively. All experiments were conducted on cells with similar passage number, ranking from 2 to 3.

#### Lactate synthesis

Lactate synthesis was estimated using enzymatic measurement of lactate concentration in an acidified incubation medium, in line with [17,18]. This allowed for determinations of both intracellular lactate content and lactate released from the fibroblast cultured in the glucose and glucose-free media. The cells were incubated for 30 min. in Krebs-Henseleit solution (10 mM HEPES, 2 mM NaHCO_3_, 135 mM NaCl, 3.5 mM KCl, 0.5 mM NaH_2_PO_4_, 0.5 mM MgSO_4_, 1.5 mM CaCl_2_, supplemented with 1 mM pyruvate and 5.6 mM glucose, pH 7.4). The incubation was next acidified with HClO_4_ (final concentration 5%). After 10 minutes on ice, extracts were neutralized with 2 M K_2_CO_3_, prior to fluorimetric lactate measurement using lactate dehydrogenase in a buffer composed of 0.5 M glycine, 0.4 M hydrazine sulphate and 5 mM EDTA, at pH 9.5. Lactate concentration was calculated by reference to a standardized lactate solution. Protein concentration was measured in cells solubilized in 0.5 M NaOH using the Modified Lowry Protein Assay Kit (Pierce). Data are presented as lactate content in nanomoles per milligram of protein.

#### Cellular respiration

Respiration was measured polarographically, with the use of the Oxygraph-2k (OROBOROS; INSTRUMENTS GmbH, Austria), as described earlier [18]. Cells from a 10 cm-diameter tissue culture dish were trypsinized and resuspended in pre-warmed PBS to final protein concentrations of 0.15–0.3 mg/ml. Oxygen consumption was measured at 37°C in substrate-free solution (PBS), and then after administration of 0.1 mg/ml oligomycin and CCCP (1μM). Following the respiration assay, a cell suspension was mixed with 0.5 M NaOH (1:1), before being made subject to measurement for protein concentration. Data are presented by reference to the mean use of O_2_ in picomoles per milligram of protein per second.

#### MitoTracker staining

Control and proband-derived fibroblasts were grown on glass coverslips in glucose and glucose-free medium for 72h. For mitochondrial staining, cells were incubated with 50 nM MitoTracker Red CMXROS (Molecular Probes) for 30 minutes at 37°C. After incubation, cells were washed twice with Phosphate Buffered Saline and fixed with 4% PFA. Images were obtained by Zeiss LSM 510 confocal microscopy. Mitochondrial morphology was examined utilizing ImageJ to analyze the index of elongation.

#### Transmission electron microscopy

Endoplasmic reticulum ultrastructure was evaluated by means of transmission electron microscopy. Controls and proband-derived fibroblasts grown in a glucose or glucose-free medium for 72 hours were fixed in 1% paraformaldehyde and 1.25% glutaraldehyde in cacodylate buffer, at pH 7.4 for 20 h, before being postfixed in a mixture of 1% osmium tetroxide (OsO_4_) and 0.8% potassium ferricyanide K_4_[Fe(CN)_6_]. The material was then processed for transmission electron microscopy using standard protocols, and analyzed in JEOL 1011.

#### Mitochondrial DNA content

Mitochondrial DNA content was measured using real-time PCR, in line with the protocol published by Zsurka, 2008. Quantification was based on the measurement of quantities of mtDNA relative to nuclear DNA (Kir4.1). All primers and probes used in the current study were adopted from Zsurka [19]. In brief, mtDNA was detected using primers MT16520F and MT35R, in the presence of probe MT16557TM. Nuclear DNA content was estimated by amplification of a fragment from the single-copy gene Kir4.1, with primers KIR835F and KIR903R used together with probe KIR857TM. Reactions were performed using the Applied Biosystems® 7500 Real-Time PCR System (Life Technologies). Total DNA was extracted from control and proband-derived cells grown in glucose and glucose-free medium for 72 hours using an E.Z.N.A.® MicroElute Genomic DNA Kit (Omega Bio-tek). Relative mtDNA quantity was determined using the ΔΔCt method.

#### Western blotting

Cell extracts were prepared with Lysis Buffer (Cell Signaling), separated by 10% SDS-polyacrylamide gel electrophoresis (20 μg of protein per line) and electro-transferred to nitrocellulose membrane. Membranes were blocked with 5% milk in tris-buffered saline, at pH 7.6, containing 0.05% Tween 20 (TBST), incubated with primary antibody recognizing GRP78/BiP protein (Abcam, 1:1000 in TBST) at room temperature for 2 hours, and next with horseradish-peroxidase–linked secondary antibody (Sigma, 1:8000 in 5% (w/v) milk in TBST), or with anti-β-Tubulin directly linked with HRP (Abcam, 1:10000 in TBST), for loading control. The bands were detected by ECL using Fusion Fx (Vilber Lourmat), and then quantified by densitometry.

#### Statistics

Data are shown as mean value ± standard deviation. Statistically significant differences were calculated using a Student t-test.

### Molecular modelling

The molecular structure of the human MFN2 protein was obtained through the application of the homology modelling approach using the I-TASSER server [20], with the bacterial dynamin-like protein (BDLP, PDB:2J69) as a template. That homologue protein contains a full-length structure with only three fragments missing, i.e. 3 N-terminal amino acids and two gaps in the HR2 domain (8 and 13 amino acids) [21]. Residuals 595 to 635 were obtained unstructured, and the corresponding BDLP region was folded into an μ-helix. Manual refinement was thus required, and folding of the region into an α-helix was achieved using the YASARA software package [22,23], with the YASARA force field [24]. The MFN2 homodimer was constructed by reference to the 2J68 PDB structure [21]. This homodimer model is consistent with the suggested complex orientation after membrane fusion [25]. The protein contains regions anchored to the membrane—a lipid bilayer was prepared with the CHARMM-GUI Membrane Builder [26,27] using POPC, POPE and SAPI lipids at ratios corresponding to the normalized composition of the outer mitochondrial membrane (51.58%, 36.88% and 11.54%, respectively) [28]. The initial membrane structure was subject to 0.5 ns of molecular dynamics simulation, resulting in hydrophobic lipid tail melting. Simulation parameters are as given below. Missing topology and force field parameters for simulations were obtained from the CgenFF server v. 0.9.7.1 beta [29,30]. Stability of the model structure was confirmed running 5 to 20 ns of molecular dynamics simulations. Stability in the 2.5 to 15 ns time-windows was observed. All simulations were carried out at T = 310 K, under atmospheric pressure, and with 0.05 M ionic strength in a rectangular cuboid. The NAMD library [31] capable of carrying out simulations for large-molecular systems with scalable parallel MD algorithms was used. The CHARMM27 force field [32–38] and TIP3P water model were applied.

The GTP molecule was docked in the GTPase binding site using MOE docking technologies [39], with the MMFF94 forcefield but without a conformational search, since the localization of the ligand and its conformation were known from the 2J68 PDB entry. The resulting binding modes were scored and relaxed using energy minimization. Using these results, diamond-shaped dimeric MFN2 structures containing the ligand in one subunit were built. Structural stability of the most probable complexes in water solution were tested by 3 to 9 ns molecular dynamics simulations, as run with careful thermalization and equilibration of systems. Structures remained stable, and Arg274Gln and Arg274Trp substitutions were introduced into both subunits prior to the repetition of molecular dynamics simulations. The MM-GBSA approach considering selected snapshots from trajectories after system relaxation was applied to estimate free energies of binding [40].

#### Assay for GTPase activity

The postnuclear supernatants (PNS) from control and proband-derived fibroblasts were obtained after Ishihara *et al*. 2004 [41]. Cells were homogenized in 10 mM HEPES-KOH buffer (pH 7.4) containing 0.22 M mannitol, 0.07 M sucrose and protease inhibitor cocktail (Sigma), and then centrifuged at 200 g for 5 minutes to remove nuclei.

GTP hydrolysis in control and proband fibroblasts was analyzed using the approach described by Ishihara *et al*. 2004 [41], with some modifications. Endogenous MFN2 was purified from PNS (300 μg) by an immuprecipitation method using the anti-MFN2 antibody (Sigma). The purified proteins were incubated with 40 μl of 20 mM HEPES-KOH (pH 7.4), containing 2 mM MgCl_2_, 1 mM DTT, 1% Triton X-100 with 100 μM GTP, at 37°C for 1 h. The reaction mixture was removed and analyzed by HPLC. The samples were applied to an IonPac AS11-HC column, preceded by IonPac AG11-HC, at 30°C in a KOH gradient (1–80 mM), at a flow rate of 0.38 ml/min with detection at 260 nm (Dionex ICS 3000). In this system, GDP elutes at 40.6 min and GTP at 43.9 min.

## Results

### Case report

The (male) patient was born after an uneventful pregnancy and labor, with a birth weight of 4100 g and length of 56 cm, and 10 points on the APGAR scale. He sat at 7 months, and start to walk at 11 months. He manifested first disease symptoms at the age of 10 years (trouble in walking). Despite his mild mental retardation, he has well-developed social abilities. However, he has weak reasoning, abstract and mathematical thinking, and needs the help of another person to solve any problems. His visual abilities are on the low border of the norm.

**Fig 1:**
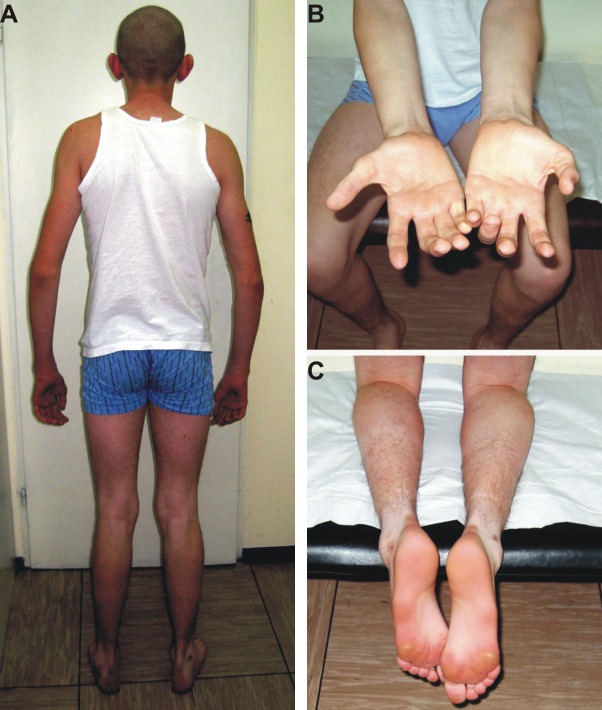
Clinical characterization of the CMT2A patient harboring the p.Arg274Trp mutation. A. Wasting of distal muscles (upper and lower limbs) in the proband. B. Severe wasting of the small hand muscles and forearms in the proband. C. *Pes cavus* deformity in the proband.

On neurological examination at the age of 23 years, he presented with symmetrical muscle atrophy in the distal parts of both the upper and lower limbs (Fig 1). Additionally, the proximal muscles of his lower limbs showed atrophy. The cranial nerves were normal. Ankle jerks were absent and *pes cavus* deformity present (Fig 1C).

Neurographical examination (ENG) was performed at the age of 16 years. In the motor fibers of the median nerve, nerve conduction velocity (NCV) was markedly reduced (15 m/sec), while the compound muscle action potential amplitude (CMAP) was also severely reduced (0.8 mV). CMAP was also severely reduced in the peroneal and tibial nerves (0.0 and 0.1 mV), respectively. Sensory nerve conduction velocity (SNCV) in the median nerve was slightly reduced (33.3 m/s), while sensory nerve action amplitude (SNAP) was markedly reduced (0.75 μV). The results of standard laboratory tests were within the norm.

The Sanger sequencing revealed a heterozygous *de novo* c.820C>T (p.Arg274Trp) mutation in the patient’s mitofusin 2 [1]. The parents of the proband are healthy, however, and there is no evidence of consanguinity within the family, though the parents do originate from the same village.

In summary, the proband presented with a moderate, early-onset motor-sensory polyneuropathy. Both axonal and demyelinating features of the neuropathy were observed in nerves of the upper and lower limbs. There was also proximal-muscle involvement in the lower limbs. Additionally, the patient manifested mild mental retardation.

### Whole exome sequencing and data analysis

The whole exome sequencing (WES) approach was taken, with a view to the molecular background of the proband’s complex phenotype (of moderate peripheral neuropathy and mental retardation) being understood. We thus attempted to identify other mutations acting additionally to the main and causative *MFN2* gene mutation.

Whole exome sequencing of proband DNA confirmed the c.820C>T (p.Arg274Trp) mutation in the *MFN2* gene, as identified earlier using the Sanger method. No variants in known genes associated with mental retardation were found. Moreover, an absence of pathogenic or possibly deleterious sequence variants in the *POLG* gene was revealed.

Additionally, we selected 113 genes (S1 Table) in which mutations have been shown by others to be causative (according to the Pubmed and OMIM databases) for hereditary neuropathies (sensory, motor and sensory-motor) [16]. In parallel, mitochondrial genes were analyzed in the same way, taking 98% as mean coverage.

Data analysis of proband WES results for gene hereditary neuropathy and mitochondrial genes gave 74 variants, of which three showed a likely effect on patient phenotype. Of the latter, only one homoplasmic mutation in leukocytes in *MT-CO1* – m.7444G>A has been described in a patient and designated as pathogenic–in Leber hereditary optic neuropathy (LHON), with sensorineural hearing loss and maternally inherited deafness or aminoglycoside-induced deafness. The heterozygous c.385A>G p.Met129Val in the *PRNP* gene has been described as a risk factor for prion disease, early-onset Alzheimer disease and primary progressive aphasia. The *WNK1* gene heterozygous variant c.2175dupC p.Ile726Hisfs occurring with unknown frequency is supposed to have a pathogenic effect (in hereditary sensory and autonomic neuropathy type IIA), in its homozygous state.

None of the above symptoms were observed in our patient. Moreover, analysis of all data by allele frequency (≤ 2%) and possible impact on proteins (predicted by *in silico* tools (SnpEff and SnpSift-Galaxy Platform) revealed 419 rare variants. In the case of genes associated with human disorders, we rejected variants occurring in the control healthy group in the homozygous state, for variants in genes inherited in a recessive mode and heterozygous for variants in genes that are inherited in a dominant trait.

### Morphology, lactate formation and cellular respiration

**Fig 2:**
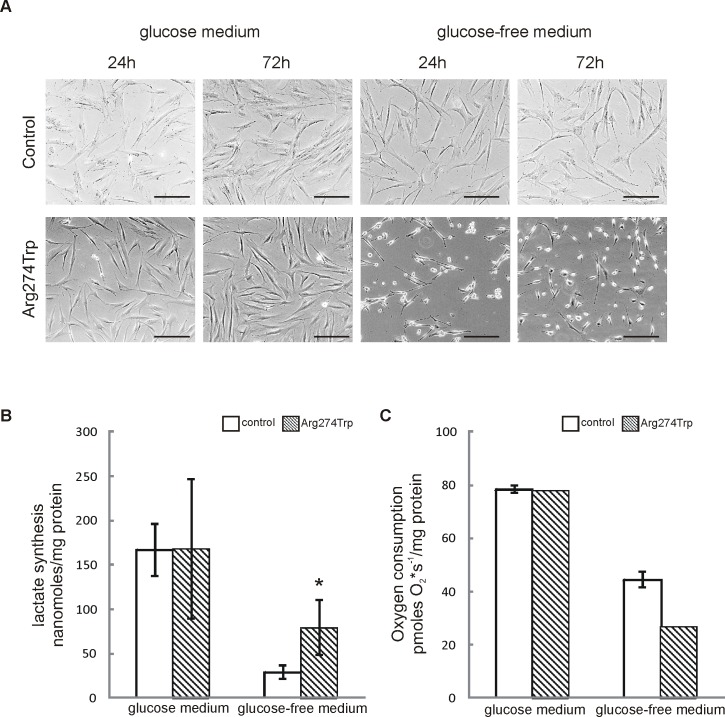
Morphology, lactate formation and oxygen consumption in proband-derived fibroblasts A. Transmitted light images of control and proband-derived fibroblasts cultured for 24 and 72 hours in glucose and glucose-free medium. Bar = 200 μm. B. Lactate synthesis was measured enzymatically in control and proband-derived fibroblasts cultured in glucose and glucose-free medium for 72 h. Data are expressed as mean amount of lactate (nanomoles per mg of protein) ± S.D.; n = 3, *p < 0.05. C. Oxygen consumption of cells grown in glucose or glucose-free media for 72 h was measured polarographically at 37°C. Data are expressed as pmoles O_2_ x s^-1^/mg protein and show mean values ± S.D.; n = 2–3.

Early passages (2–3) of control and proband-derived fibroblasts were observed for 24 and 72 hours in glucose and glucose-free medium. No differences in morphology and growth rate between control and proband-derived fibroblasts in glucose medium were observable (Fig 2A). Upon exposure to the glucose-free condition, most of the cells harboring the *MFN2* mutation took on a round shape, as well as rates of proliferation that were lower than in control cells, both 24 and 72 hours into culture. This reduction in proliferation might be the effect of oxidative phosphorylation impairment occurring in glucose-free medium among mutation-affected cells, with this being demonstrated as high lactate formation and low oxygen consumption (Fig 2B and 2C). In glucose-free medium, intact cells are forced to rely on oxidative phosphorylation for energy production [42], with the result that levels of lactate in the cells are low. As Fig 2B and 2C show, proband cells cultured in glucose-free medium maintain a high level of glycolytic metabolism, with the result that lactate levels are higher, and oxygen consumption lower, than in control cells.

### Mitochondrial and endoplasmic reticulum morphology

**Fig 3:**
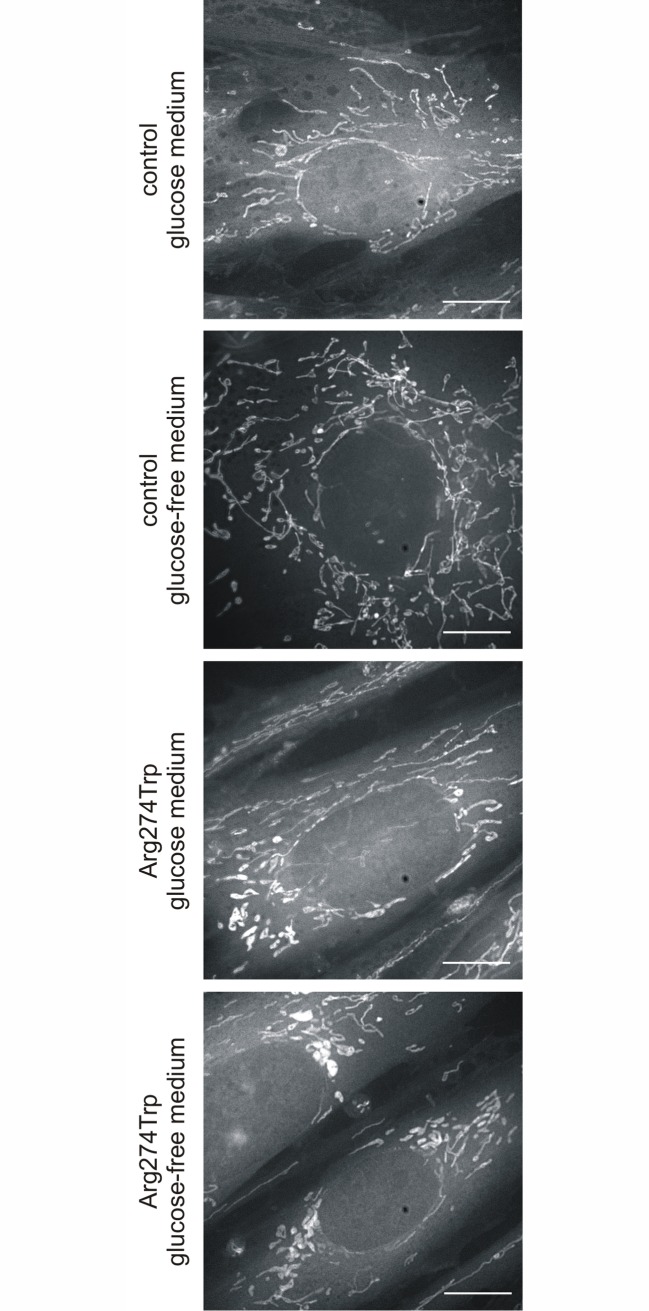
Mitochondrial morphology in control and proband-derived fibroblasts cultured for 72 hours in glucose and glucose-free medium. Fibroblasts were stained with 50 nM MitoTracker for 30 min in 37°C and visualized using confocal microscopy. Representative images of MitoTracker stained mitochondria are shown. Bar = 10 μm.

MitoTracker staining revealed that proband-derived cells had an altered mitochondrial network, as well as altered distribution of mitochondria and their transport to fibroblast protrusions. As Fig 3 shows, 72 hours into the exposure to the glucose-free condition, mitochondria with a perinuclear location are fragmented. 60% of mitochondria appeared mostly as spheres or ovals, while tubules were in the minority and presented by only 40% of mitochondria. Changes in mitochondrial morphology were already evident after 24 hours in a glucose-free medium (data not shown), and in subsequent days the effect became more and more pronounced. The parallel, control fibroblasts mostly contain tubular mitochondria, distributed in a roughly radial manner throughout the cytoplasm. In the glucose medium the presence of tubules characterized 80% of mitochondria, while in the glucose-free medium the number of elongate mitochondria decreased in favor of the oval, with these respectively accounting for 67% and 33% of the numbers.

**Fig 4:**
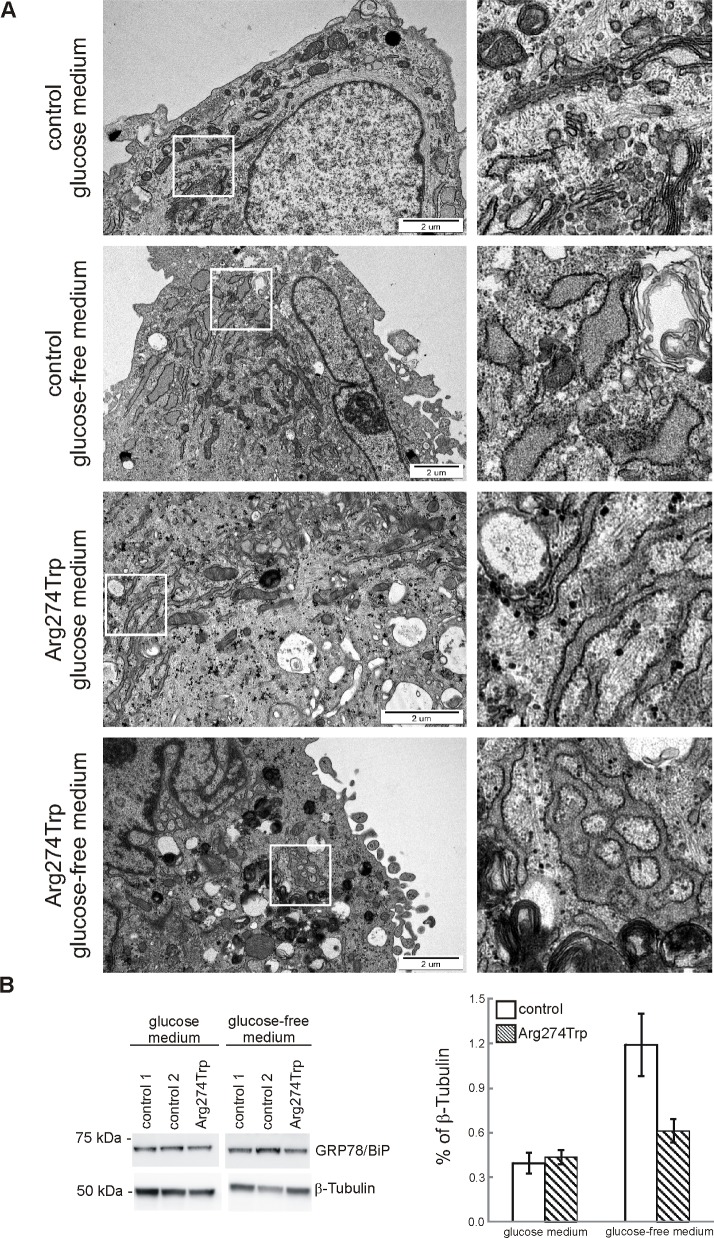
Endoplasmic reticulum morphology and reticulum stress in control and proband-derived fibroblasts cultured for 72 hours in glucose and glucose-free medium. A. Representative transmission electron microscopy images of ultrastructure of endoplasmic reticulum (ER) are shown. On right, zoomed pictures to highlight distinct ER structures. Bar = 2 μm. B. Immunoreactivity of reticulum stress marker, GRP78/BiP, was analysed by western blot. Densities of GRP78/BiP bands were evaluated and data are expressed as a percentage of β-Tubulin. (mean ± SD, n = 2–3).

On the basis of data indicating linkage between MFN2 and the biology of the endoplasmic reticulum (ER) [43], we examined the effect on ER morphology of culturing control and proband-derived fibroblasts in glucose and glucose-free media (Fig 4). Cells were cultured for 72 hours in both kinds of media, and then processed for TEM visualization. Under glucose conditions, the comparison with control fibroblasts revealed a major morphological class encompassing >55% of proband-derived cells and showing evident ER expansion and enlargement, characterized by multilamellar structures (Fig 4). In contrast, only a minority, of between 14 to 20%, of control cells contained a network of slightly enlarged ER cisterns. The glucose-free condition, regarded as causing ER stress [44] led to notable ER enlargement in controls, encompassing >80% of cells; while aggravating pathological morphology in proband-derived cells, among which more than 90% of cells had dramatic broadening and fusion of the endoplasmic reticulum. Furthermore, western blotting analysis showed marked enhancement of GRP78/BiP in control cells, while in proband-derived cells the changes were less profound as compared with glucose-containing cultures. As this effect was obtained in the course of prolonged ER stress, in control cells it rather plays a role in some metabolic adaptive response, while in proband-derived cells whose mitochondria are dysfunctional, it reflects an impaired unfolded protein response. The latter is in line with data showing that cells with impaired mitochondria display increased toxicity in response to glucose deprivation, with this sensitivity linking up to an impaired unfolded protein response [45].

### Mitochondrial DNA content

**Fig 5:**
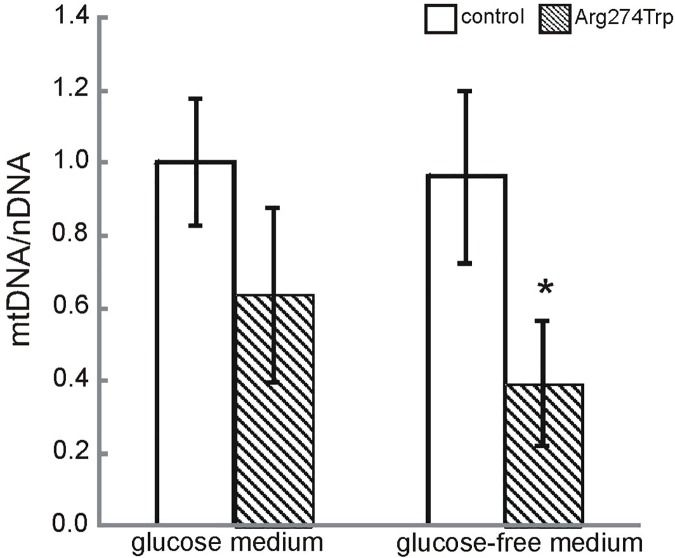
Mitochondrial DNA content was determined in control and proband-derived fibroblasts cultured for 72 hours in glucose and glucose-free medium by real time PCR. Quantification was based on mtDNA to nuclear Kir4.1 (nDNA) gene encoding ratio. Mean values and standard deviations were calculated from at least X independent experiments. *p<0.05

To verify if mitochondrial dysfunction described in the patient-derived fibroblasts could be attributed to reduced mitochondrial DNA content, we examined mitochondrial to nuclear DNA ratio in control and proband-derived fibroblasts cultured for 72 hours in glucose and glucose-free medium (Fig 5). Relative mtDNA levels were not found to differ significantly between control and proband-derived cells in glucose conditions. However, the replacement of glucose by glucose-free medium was associated with an mtDNA/nDNA ratio in proband-derived cells almost 60% lower than in the control (at 0.393 ± 0.172, n = 8, p < 0.05). Taken together, the above data point convincingly to a mitochondrial involvement in proband pathology observed in fibroblasts cultured even in glucose medium, with this becoming accented strongly in a glucose-free medium. These results are not consistent with data published previously, which failed to observe mitochondrial dysfunction in patient-derived fibroblasts [46]. However, we believe that the early passages of patient-derived fibroblasts plus a (glucose-free) medium forcing oxidative phosphorylation are crucial if mutated mitofusin 2-induced abnormalities in fibroblasts are to be revealed.

### Molecular modeling

**Fig 6:**
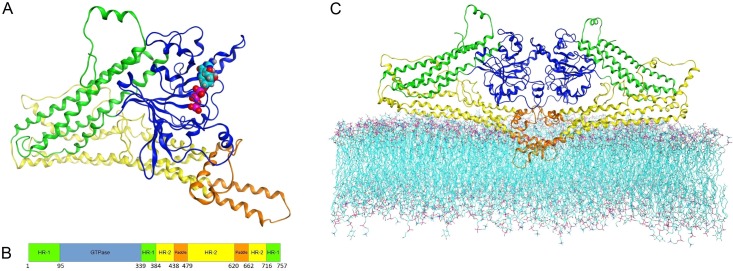
Overall MFN2 model structure. Colors demonstrate domain division: GTPase (blue), HR-1 (green), HR-2 (yellow), and paddle region (orange). A. MFN2 monomer structure with visible GTP showed as Van der Waals spheres. B. Sequence-oriented MFN2 domain composition, with boundary residues. C. MFN2 homodimer embedded in lipid bilayer. Paddle residues 620–662 deeply embedded, residues 438–479 localized above hydrophobic layer.

A member of the large group of proteins with a GTPase domain, the MFN2 protein is characterized by some conserved sequences in the GTP-binding domain, and by common structure. It is distinguished from others members of its group by certain additional architectural features common to all members of the mitofusin family (as well as to the dynamin and BDLP families), i.e. the structure of the large GTPase domain (distinct from other GTPases) and the presence of two additional domains [47]: HR-1 (also called the Neck or middle domain) and HR-2 (also called the Trunk or GED—GTPase effector domain), which contain the important transmembrane part known as the paddle region. The structural model of MFN2 proposed here includes all of these domains (Fig 6A and 6B).

**Fig 7:**
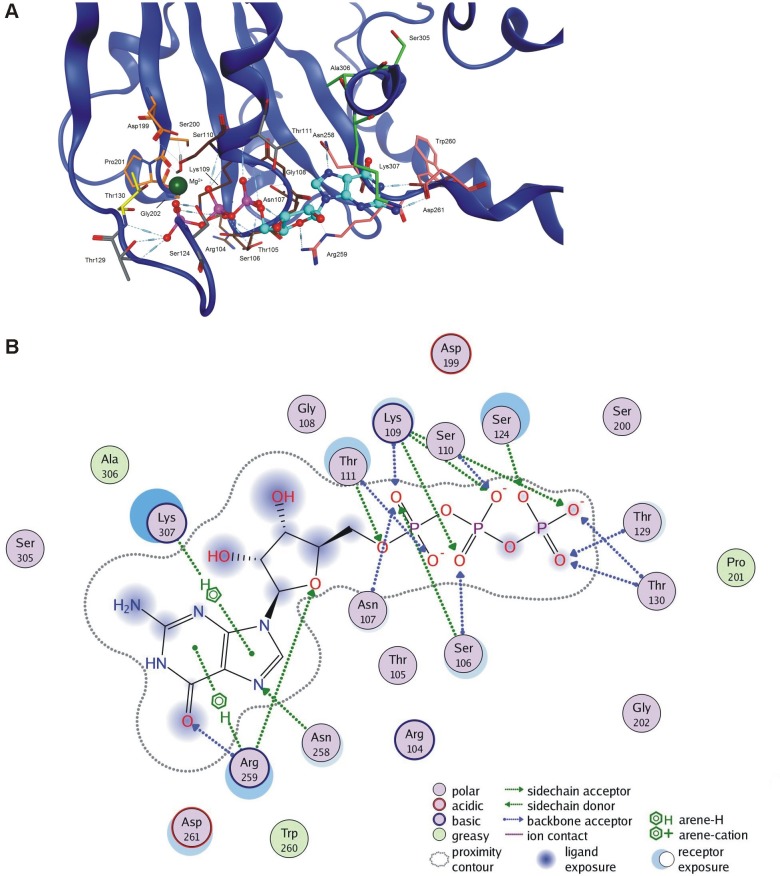
MFN2 GTP binding site and interactions. A. 3D structure of GTPase pocket with GTP ligand present. Oxygen atoms depicted in red, nitrogen in blue, phosphorus in purple. Carbon colors represent specific regions: G1 (brown), G2 (yellow), G3 (orange), G4 (mauve), G5 (green) and additional interacting residues (grey). Ligand carbons are cyan-colored. B. 2D scheme of GTP interactions with the MFN2 pocket.

The GTPase domain is a highly-conserved structure in the mitofusins (most especially at the GTP-binding site), with responsibility for the binding and hydrolysis of GTP. The domain relates structurally to similar ones in dynamin and BDLP. It contains an 8-stranded β-sheet surrounded by seven α-helices, within which the five important motifs G1-G5 may be identified, with these also being found in other members of the GTPase protein superfamily [48]. The P-loop (G1) motif (GxxxxGKS/T, ^103^GRTSNGKS^110^ in MFN2) functions as a coordinator of phosphate groups of the bound nucleotide. A conserved threonine (Thr130 in MFN2) in switch-I (G2), and conserved residues DxxG (^199^DSPG^202^ in MFN2) of switch-II (G3) are involved in Mg(2+) binding and GTP hydrolysis. G1, G2 and G3 are further highly conserved motifs that together form a catalytic center; while G4 and G5 motifs ensure specificity for GTP binding [48]. A role in base binding is ascribed to the G4 motif (usually N/TKxD, though mitofusins contain R instead of K) [49]. The side chain of Asn258 interacts with the base through the hydrogen bond, as expected. In our model, Arg259 interacts with oxygen in the endocyclic ribose–it is the same interaction as that observed in the case of conserved lysine in related proteins. The side chain of Asp261 makes two hydrogen bonds to the nucleotide. The G5 (SAK/L) or G-cap motif is involved in binding the ribose moiety. It is present in the structure (^305^SAK^307^ in MFN2), though that occurrence has not been tested in mitofusins, hence the frequent exclusion of the G5 motif from further consideration (Fig 7A).

On the basis of results for GTP docking into MFN2, it has proved possible to identify amino acids from the GTP binding site. Those usually participating in ligand binding, and having a direct effect on GTP binding are from motifs G1 (especially 105–110), G4 and G5. Additionally, we observed some frequent interactions with the phosphate part of the ligand formed by side chains of Thr111, Ser124, Thr129, as well as backbone interactions created by Thr111, Thr129 and Thr130 (Fig 7A and 7B). Thr111 is also able to form a hydrogen bond with the guanosine part of the ligand.

Both the HR-1 and HR-2 domains consist of three long α-helices arranged in parallel, as well as two shorter helices each. On the borderline between the HR-1 and HR-2 domains there are structural hinges allowing for a degree of movement. This kind of structure suggests that conformational changes are readily induced by the binding of a GTP molecule, and/or by GTP hydrolysis.

The aforementioned, so-called paddle region contains three α-helices arranged perpendicularly to each other. Two of these (residues 620–661) form an extension of the helix of the HR-2 domain, giving rise to a complete rigid structure. Moreover, the paddle domain is inserted into a lipid bilayer. This suggests that the paddle region of MFN2 is likely to have a direct involvement in membrane fusion.

The structure described was used to construct a diamond-shaped homodimer similar to the one formed by BDLP [21]. The modelled complex remained stable in molecular dynamics simulations. The model of the MFN2 dimer embedded in the membrane is shown in Fig 6C. The maximum distance between the MFN2 paddles and the top of the GTPase domains is of about 8.0 nm, a distance similar to that in BDLP dimers. Two monomers of MFN2 interact with each other through the GTPase domains, and also through transmembrane helices creating an interface across 6.28% of the monomer surface (with a 3.56% contribution from the paddles, and a 2.56% contribution from the GTPases). The overall value is lower than in the template structures, because MFN2 contains additional N-terminal amino acids increasing the solvent accessible area. GTP-binding sites are oriented towards the symmetry-mated GTPase domain and occluded, yet they are not directly opposite each other. The GTPase domains of the MFN2 monomers are bridged by hydrogen bonds between Glu230 and Arg259, and Ser263 and Lys307, as well as by electrostatic interactions between Glu230 and Arg259, and Asp170 and Lys238. The paddle helices form two hydrophobic intramolecular contacts: Val445 –Val457 and Trp631 –Ile634, as well as one symmetrical hydrogen bond: Gln449 –Gln449. Additionally, the Gln172 –Tyr448 hydrogen bond is created between the GTPase domains and the paddles.

Our model reveals that the 262–266 loop and adjacent 267–285 α-helix of the second mer covers the access to the binding site cavity. It is also important that the loop is flexible enough to create ligand interactions, namely hydrogen bonds with Ala262 and Ser263 (the sidechain of Ser263 and the backbone of Ala262-Ser263 interact with the guanosine fragment of GTP). Rare side-chain interactions of Gln276 and Arg280 with ligand phosphates were also observed. Since the diamond-shaped complex is C2-symmetrical, the above property applies to both proteins.

The discussed mutations are located in the GTPase domain, in the α-helix, contributing to the cover of the cavity referred to previously. The 274 residue under consideration is located in the closer to symmetry-mated part of the α-helix, with its side chain oriented towards the monomer interior; and it interacts with the 262–266 loop. Since no specific significant interaction with Arg274 was observed, and the site consists of amino acids demonstrating diverse properties, enough space is provided for the side chain, and substitutions do not trigger noticeable disturbances in structure. The p.Arg274Gln mutation does not influence remote intermolecular interactions, and the p.Arg274Trp mutation changes some short range Van der Waals interactions with neighboring aromatic residues, but both side chains remain in their native positions.

### MFN2 GTPase activity

**Fig 8:**
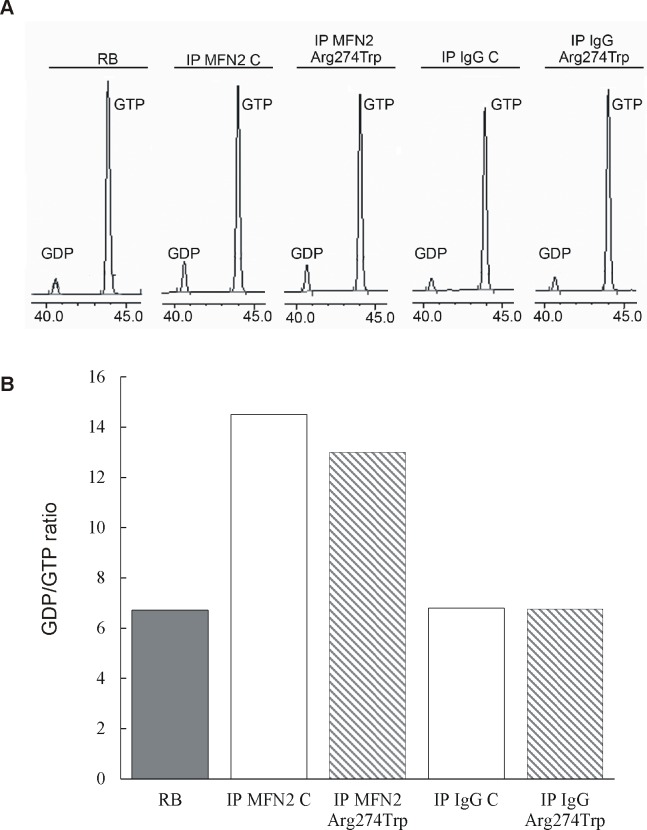
GTP-hydrolysis activity of mitofusin 2 obtained from control and proband-derived fibroblasts. GTPase activity of control and mutated MFN2 presented as HPLC traces (A) and GDP/GTP ratio (B). Endogenous MFN2 was immunoprecipitated from 300 μg PNS lysates obtained from control (IP MFN2 C) and proband-derived fibroblasts (IP MFN2 Arg274Trp) and incubated with GTP at 37°C for 60 minutes, before HPLC analysis as described in Methods. In parallel, immunoprecipitation with nonspecific rabbit antibody was performed with PNS lysates obtained from control (IP IgG C) and proband-derived fibroblasts (IP IgG Arg274Trp). RB–reaction buffer containing 100 μM GTP.

The results of molecular modelling did not point to an effect of p.Arg274Trp mutation on mitofusin 2 GTP-binding and hydrolytic activity. To confirm these data, we studied GTP- hydrolysis activity of MFN2 isolated from control and proband-derived fibroblasts. The GTP hydrolysis assay shows no difference in activity between the studied groups (Fig 8). As measured by HPLC, the mutated MFN2 generated 9.45 μM of GDP; while MFN2 isolated from control cells produced 11.23 μM of GDP. For comparison, in samples whose control and proband-derived fibroblasts proteins were precipitated with normal rabbit IgG (control: IP IgG C and proband: IP IgG Arg274Trp) the amount of GDP was much the same as in the reaction buffer (5.98 μM, 5.53 μM and 6.70 μM, respectively).

## Discussion

Mitofusin 2 is a large nuclear-encode protein located in the outer mitochondrial membrane, and involved in mitochondrial fusion. Mitochondrial fusion is known to depend greatly on the GTPase activity of mitofusin 2. *MFN2* mutation within the GTPase domain causes mitochondrial dysfunction usually accounted for by reference to impaired GTPase activity. However, there are no data to show direct dependence between the *MFN2* mutation within the GTPase domain and GTPase activity.

A problem arose with reports that two different mutations in the same codon gave rise to a different clinical outcome. We recently identified a mutation within GTPase domain of *MFN2*, p.Arg274Trp (c.820C>T), in a patient with early-onset CMT2A with peripheral axonal neuropathy, as coexisting with mild mental retardation [1]. It then emerged that a mutation in the same codon had been reported previously by Züchner, with that mutation being known as p.Arg274Gln (c.821G>A) [8]. A question therefore arose as to how these two mutations affect mitofusin 2 function, and evoke such different clinical outcomes.

The work detailed here reveals major impairment of mitochondrial and endoplasmic reticulum morphology and mtDNA content in cultured primary fibroblasts obtained from the CMT2A patient harboring the p.Arg274Trp (c.820C>T) mutation. The cellular pathological changes observed were probably affected by the presence of mutant MFN2 protein.

Next, using the stable model for mitofusin 2 obtained by us and followed by GTP hydrolysis activity assay, we revealed that the p.Arg274Trp mutation does not exert a direct impact on GTP binding. We must thus suggest that observed biological malfunctions reflect, not only impaired GTPase activity, but also other functions of mitofusin 2 (like protein-protein interactions) wherein the GTPase domain also contributes to the pathological phenotype.

The phenotype we report on in this study entails moderate peripheral axonal neuropathy and mild cognitive impairment coexisting in one patient harboring the aforesaid p.Arg274Trp (c.820C>T) mutation within the GTPase domain of mitofusin 2, [1]. Mental retardation is a constant part of the phenotype in only two forms of Charcot-Marie-Tooth disorder. In giant axonal polyneuropathy (GAN), severe mental retardation is associated with a specific phenotype of kinky hairs and giant axons with an accumulation of neurofilaments [50]. In turn, in Cowchock syndrome (CMT4X), mental retardation is associated with axonal polyneuropathy and deafness [51]. Recently, a new p.Glu493Val mutation within apoptosis inducing factor (AIF) mitochondrion associated 1 gene (AIFM1) has been identified as a causative mutation in the original Cowchock family [52]. To the best of our knowledge, the only study showing an association between *MFN2* mutation and mild cognitive impairment is that by Del Bo [53]. In 2008, he reported three CMT2-affected members of one Tuscan family harboring the c.310C>T (p.Arg104Trp) mutation within the *MFN2* gene [53]. This is located within the GTPase domain of the protein, and affects an amino acid found to be highly conserved in different species. All studied family members were affected by peripheral neuropathy, cognitive impairment and poor nocturnal vision. A member of this family also showed spastic paraparesis.

Moreover, due to the MFN2 protein being expressed within the central nervous system, some authors report on *MFN2* mutations segregating with white-matter abnormalities visualized in MRI analysis [54,55]. In some patients, *MFN2* mutations have been shown to be associated with transient dysarthria [54]. Boaretto and co-workers reported on an Italian family in which a new splice-site *MFN2* gene mutation was associated with fulminant fatal encephalopathy in three individuals [56]. The splice-site *MFN2* c.1392+2T>C mutation has been shown to generate four different splicing products most probably acting *via* a dominant-negative mechanism [3].

Given the broad genetic heterogeneity of mental retardation and the atypical phenotype of CMT2A, we performed whole exome sequence analysis in respect of our patient’s DNA, with a view to identifying a pathogenic mutation other than c.820C>T (p.Arg274Trp) that could be responsible for mental retardation. However, no variants in known genes associated with mental retardation were found. Moreover, WES revealed the absence of pathogenic or possibly deleterious sequence variants in the *POLG* gene. Data analysis of WES results for gene hereditary neuropathy and mitochondrial genes gave three variants having a potential impact on patient phenotype, i.e. the homoplasmic m.7444G>A mutation in mitochondrial DNA coding for the cytochrome oxidase I subunit (MT-COI); c.385A>G p.Met129Val in the *PRNP* gene (a major prion protein); and c.2175dupC p.Ile726Hisfs in the *WNK1* gene (the serine/threonine-protein kinase WNK1). Given to the lack of Alzheimer's or prion disease, or symptoms of aphasia in the patient, the c.385A>G p.Met129Val variant does not seem to affect the phenotype. The impact of the other two variants might be taken account of, however. To date, the m.7444G>A mutation has been identified in numerous patients suffering from aminoglycoside-induced and non-syndromic hearing loss [57]. However, a *PubMed* search did not point to any patient carrying the mitochondrial m.7444G>A mutation and manifesting mental retardation. Nor can we definitively preclude the m.7444G>A mutation impacting upon the severity of the peripheral polyneuropathy observed in our patient. In fact, some mutations resulting in Leber optic nerve atrophy manifest additionally with an axonal polyneuropathy of the peripheral nerves [58]. A similar situation exists in the case of variant c.2175dupC p.Ile726Hisfs in the *WNK1* gene, especially given reports of connections between mutations in this gene and autosomal recessive hereditary sensory and autonomic neuropathy type IIA. In the heterozygote state, this can act as a modulator, in a manner resulting in more prominent sensory symptoms.

We are thus inclined to consider a bigenic model of inheritance in our proband, leading to a cumulative effect of m.7444G>A/ c.820C>T mutations. Unlike the family reported by Del Bo, our patient did not display any symptoms of spastic paraparesis suggestive of the involvement of corticospinal tracts [53]. The p.Arg274Trp *MFN2* mutation detected by us is not causative for mental retardation alone, in contrast to the p.Arg104Trp mutation that results in sensorineural hearing impairment, peripheral neuropathy and mild cognitive decline.

*In vitro* data using proband-derived fibroblasts show a wide range of cellular pathological changes discussable in a general sense as consequences of a mutated mitofusin 2 protein. Among these are morphological abnormalities in the mitochondrial and endoplasmic reticulum network, a reduction in mitochondrial DNA content and handicapped energy metabolism. All of the above abnormalities seem to be consequences of disruptions in mitochondrial fusion, as an MFN2 GTPase activity-dependent process.

Along with mitofusin 1 and OPA1, mitofusin 2 plays a major role in the mitochondrial fusion process. MFN2 deficiency causes mitochondrial dysfunction. Hence the disruption of the mitochondrial network in proband-derived fibroblasts observed by us can come as no surprise. In the conditions of a glucose-free medium, fibroblasts had dramatically fragmented mitochondria, with the perinuclear location indicating a serious failure of transport and distribution within the cell. In the same cells, ultrastructural observation revealed evident ER expansion and enlargement, as characterized by the presence of multilamellar structures in the proband-derived fibroblasts. This observation is in line with studies reporting an association between MFN2 and the endoplasmic reticulum (ER) [59]. In the absence of MFN2, contacts between mitochondria and ER are lost, leading to calcium-homeostasis perturbations and ER-network fragmentation.

A relationship between mitochondrial fusion and mtDNA content has also been reported, so it is postulated that mitochondrial fusion could be a mechanism allowing for mtDNA maintenance through exchange of DNA molecules between mitochondria throughout the mitochondrial tubules. Disruptions of the mitochondrial network by mitochondrial fragmentation automatically would then result in reductions in mtDNA [60]. Moreover, in MEFs lacking Mfn2, Mfn1 or both, a considerable number of mitochondria are found to be devoid of mtDNA [18,61]. The reduction of mtDNA in the proband-derived fibroblast observed by us would thus seem in line with the previous study. mtDNA encodes 13 proteins of the respiratory chain, such that a depletion of mtDNA is expected to lead to impaired oxidative phosphorylation. In the Mfn2 conditional knockout mouse, a decrease in cytochrome c oxidase activity has been measured in Purkinje cells [61]. In our study, a relationship between mtDNA reduction and impaired oxidative phosphorylation was also observable. The patient-derived fibroblast culture in glucose-free medium revealed production of lactate higher than in the control, suggesting increased glycolytic activity as set against oxidative phosphorylation. In addition to reduced mtDNA content, oxidative phosphorylation impairment may result from the mutations in the cytochrome oxidase I subunit identified by WES.

To conclude, we found that mitochondrial and endoplasmic reticulum morphology and mtDNA content were affected significantly by the presence of mutant MFN2 protein. Moreover, we postulated that fibroblasts expressing CMT2A-associated *MFN2* alleles are good models for determining the pathogenic mechanism of the disease, in particular as mitochondrial fusion is studied. In our opinion, the early passages and culture of fibroblasts in a medium simulating stress conditions provides an opportunity to observe cellular abnormalities affected by the presence of mutant MFN2 protein. Moreover, patient cells allow for evaluation of mutant alleles at native expression levels, avoiding phenotypes arising from MFN2 overexpression.

Our data contrast with results indicating that, in fibroblasts, mitochondrial morphology, mtDNA content and respiratory capacity are not affected by the presence of mutant MFN2 protein [46]. Such a lack of mitochondrial dysfunction was observed in cells cultured for 4–9 passages in the low-glucose (1000 mg/L) medium supplemented with 10% FBS, where cells are still highly glycolytic, complicating the study of mitochondrial dysfunction. In our study, some of the cellular effects of MFN2 mutation were already visible where glucose (at 1000 mg/L) is present in the medium, albeit with the elimination of glucose resulting in a significant strengthening of the observed abnormalities Since cells grown in glucose-free medium rely mostly on oxidative phosphorylation to produce their ATP, they become more sensitive to factors affecting mitochondria than cells grown in a high-glucose medium [62].

Another study has shown that primary skin fibroblasts isolated from patients with mitochondrial deficiency (e.g. cytochrome oxidase deficiency, complex I deficiency, pyruvate dehydrogenase complex deficiency or multiple respiratory chain defects) were not able to survive when cultured in a galactose-based medium [63]. Thus the culture of primary fibroblasts in a glucose-free medium would seem to offer a good alternative to glucose medium where the study of mitochondrial dysfunction is concerned [42].

Molecular modelling was carried out to solve the problem of whether the molecular changes observed by us may reflect impairment of mitofusin 2 GTPase activity. Thus far, a major obstacle to the study of the effects of MFN2 mutations on GTPase activity has been its lack of stable structure. Here, we present a stable model of mitofusin 2 that takes account of all available biological and structural information.

The designed homodimer model is consistent with the suggested complex orientation following fusion of membranes [25]. The paddle regions of the two subunits are anchored to the same lipid bilayer, hence this conformation would seem to be the final stage of MFN2 fusion activity. The most protruding paddle amino acids barely reach the distant membrane layer. Paddle regions are well suited, and form a relatively large contact area for small regions, generating a stable conformation that is difficult to disassemble. Taken together with fact that the BDLP diamond complex was observed in systems without a membrane [21], this suggests that MFN2 might dissociate from the membrane, maintaining the diamond-shaped form.

It’s well known that dimerization in the dynamin superfamily, as well as in BDLP, is promoted following ligand binding. Regardless of that, the BDLP homodimer was observed in the absence of the nucleotide. It had a limited interface (3.3% vs. 7.3% of the monomer surface), which means that the nucleotide-free and nucleotide-bound dimers present different structure [21]. It is also unclear if, during dimer formation both MFN2s require both bounded ligands, or only one of them. Independently, our model shows that there is enough space for the GTP binding by both mitofusins, and that those ligands would not interfere with each other in that particular shape of the dimer. Structural analysis of their binding sites reveals that amino acids contributing to ligand binding are in contact with the other GTPase structure. This may indicate that, as in the case of DynA (as a member of the dynamin family), GTP-binding to both GTPase domains is crucial for hydrolysis [64]. MFN2 residuals (259 to 263) forming loops containing parts of the G4 motif of the opposing subunits, remain in close proximity, and amino acids 263 to 269 interact with the symmetry-mated α-helix 307 to 315. Amino acids 105 to 108 are also placed close to the Glu230, Ser231 and Met234 residuals of other subunits. Two former symmetry-mated unit regions are placed in close proximity to the guanosine part of the ligand, the latter to the phosphate part. The exact moment and purpose of GTP hydrolysis along the MFN2 activity path is yet to be elucidated [49]. We hypothesize that those three regions might take part in cooperative GTP hydrolysis and represent the first step in the homodimer formation process. Our structure shows that GTPase binding sites are occluded, and that ligand approach or release is highly unlikely in this structure. However, it is not impossible, since we observe a small cavity between the 307–315 α-helix and the 122–128 loop of the binding subunit, as well as the 267–284 α-helix of the other one.

Mutations p.Arg274Trp and p.Arg274Gln located in the GTPase domain do not alter the ligand association to the MFN2 binding site. Firstly, the residue under consideration is not in direct contact with GTP. The distance between the Cα atom and the closest ligand non-hydrogen atom is in the 19.0–23.5 A range, and does not depend on mutation. That range appears to be a result of the residue being located on a relatively flexible α-helix protruding from the rest of the GTPase structure. The mobility of that helix allows for interactions with the adjacent loop described above. Secondly, we have computed binding free energies in the ligand-protein complexes, confirming that there is no significant difference between the wild-type and mutated systems. The energy for the wild-type complex equals -34.47 +/- 3.62 kcal/mol. In the mutated complexes it is -31.90 +/- 4.05 kcal/mol and 34.01 +/- 4.84 kcal/mol, for p.Arg274Gln and p.Arg274Trp, respectively. The results are equal within the margin of statistical error, demonstrating the absence of an influence on direct ligand binding due to mutation. However, since a biological effect of these substitutions has been observed, we suggest that the observed malfunctions are not a consequence of impaired ligand binding or hydrolysis, but rather arise from some other MFN2 activities involving that region, e.g. interactions with other proteins. A similar mutation, p.Arg104Trp, is located in the P-loop in a different environment. The Arg104 side chain is oriented towards the solvent and does not take part in direct GTP binding. In the case of the p.Arg104Trp mutation, that residue would tend to limit its contact with water, and is likely to orientate inward in the protein molecule. Since it belongs to the G1 motif, we can assume that that rearrangement causes the P-loop deformation, which might impair GTP binding and hydrolysis. This is one plausible cause, yet particular MFN2 mechanisms driving cellular functions remain poorly understood. Exact moments of possible MFN2 conformational change are still unknown. Precise interactions with other proteins are not well characterized either. As a consequence, we still cannot state which function of MFN2 is impaired by Arg274 substitutions. What is known for sure is that this is not caused by direct GTP binding and GTP hydrolysis activity, what we also tested on patient-derived fibroblasts.

## Conclusions

We revealed that MFN2 mutations within the GTPase domain often fail to result in impaired GTP binding and GTPase activity. Observed biological malfunctions caused by MFN2 mutation within the GTPase domain would therefore be rather a consequence of an impaired contribution of the GTPase domain in other MFN2 activities involving that region, for example in protein-protein interactions.

We suggest that both the mtDNA/nDNA ratio and silico modelling are good methods of value in assessing the pathogenicity of a given mutation in MFN2. At present, whole exome sequencing methods may result in the identification of a series of mutations located in CMT-genes. There is thus a need to evaluate a potential pathogenic effect of MFN2 mutations using functional and bioinformatics-derived tools.

## Supporting Information

S1 Table The 113 genes associated with peripheral nerves disturbances, analyzed in the WES data from the patient. (DOCX)

## Abbreviations

CMT2A: Charcot-Marie-Tooth disease type 2A
MFN2: mitofusin 2
mtDNA: mitochondrial DNA
GTP: Guanosine-5'-triphosphate
WES: whole exome sequencing
POPC: 1-palmitoyl-2-oleoylphosphatidylcholine
POPE: 1-palmitoyl-2-oleoylhosphatidylethanolamine
SAPI: 1-stearoyl-2-arachidonylphosphatidylinositol
ER: endoplasmic reticulum
TEM: transmission electron microscopy
