# Supplementary material for: The Effect of a Novel c.820C>T (Arg274Trp) Mutation in the Mitofusin 2 Gene on Fibroblast Metabolism and Clinical Manifestation in a Patient
Source: PLoS One. 2017 Jan 11;12(1):e0169999. doi: 10.1371/journal.pone.0169999 (PMC5226824; doi:10.1371/journal.pone.0169999)
Supplement: S1 Table — (DOCX) [file pone.0169999.s001.docx]

Supplementary Table 1 The 113 genes associated with peripheral nerves disturbances, analyzed firstly in the WES data from the patient.

| **Gene symbol** | **Gene name** | **Reference sequence number** |
| --- | --- | --- |
| *AARS* | alanyl-tRNA synthetase | NG_023191.1; NM_001605.2 |
| *ABHD12* | abhydrolase domain containing 12 | NG_028119.1; NM_001042472.2 |
| *AIFM1* | apoptosis inducing factor, mitochondria associated 1 | NG_013217.1; NM_004208.3 |
| *ARHGEF10* | Rho guanine nucleotide exchange factor 10 | NG_008480.1; NM_014629.3 |
| *ARSA* | arylsulfatase A | NG_009260.2; NM_000487.5 |
| *ASAH1* | N-acylsphingosine amidohydrolase 1 | NG_008985.1; NM_177924.4 |
| *ATL1* | atlastin GTPase 1 | NG_009028.1; NM_001127713.1 |
| *ATL3* | atlastin GTPase 3 | NG_033985.1; NM_015459.4 |
| *ATP7A* | ATPase copper transporting alpha | NG_013224.2; NM_000052.6 |
| *BICD2* | BICD cargo adaptor 2 | NG_033908.1; NM_000052.6 |
| *BSCL2* | BSCL2, seipin lipid droplet biogenesis associated | NG_008461.1; NM_032667.6 |
| *C12ORF65* | chromosome 12 open reading frame 65 | NG_027517.1 ; NM_001143905.2 |
| *CCT5* | chaperonin containing TCP1 subunit 5 | NG_012160.1 ; NM_012073.4 |
| *CLTCL1* | clathrin heavy chain like 1 | NG_033805.1; NM_007098.3 |
| *COX6A1* | cytochrome c oxidase subunit 6A1 | NG_034299.1; NM_004373.3 |
| *CTDP1* | CTD phosphatase subunit 1 | NG_007988.1; NM_004715.4 |
| *DARS* | aspartyl-tRNA synthetase | NG_034149.1; NM_001349.3 |
| *DCAF8* | DDB1 and CUL4 associated factor 8 | NG_034154.1; NM_015726.3 |
| *DCTN1* | dynactin subunit 1 | NG_008735.2; NM_004082.4 |
| *DHH* | desert hedgehog | NG_008973.2; NM_021044.3 |
| *DHTKD1* | dehydrogenase E1 and transketolase domain containing 1 | NG_033248.1; NM_018706.6 |
| *DNAJB2* | DnaJ heat shock protein family (Hsp40) member B2 | NG_029553.1; NM_001039550.1 |
| *DNAJC3* | DnaJ heat shock protein family (Hsp40) member C3 | NG_041830.1; NM_006260.4 |
| *DNM2* | dynamin 2 | NG_008792.1; NM_001005360.2 |
| *DNMT1* | DNA methyltransferase 1 | NG_028016.3; NM_001130823.2 |
| *DRP2* | dystrophin related protein 2 | NG_016403.1; NM_001939.2 |
| *DST* | dystonin | NG_029322.2; NM_001723.5 |
| *DYNC1H1* | dynein cytoplasmic 1 heavy chain 1 | NG_008777.1; NM_001376.4 |
| *EGR2* | early growth response 2 | NG_008936.2; NM_000399.4 |
| *FAM134B* | family with sequence similarity 134 member B | NG_016644.2; NM_001034850.2 |
| *FBLN5* | fibulin 5 | NG_008254.1; NM_006329.3 |
| *FBXO38* | F-box protein 38 | NG_033871.1; NM_030793.4 |
| *FGD4* | FYVE, RhoGEF and PH domain containing 4 | NG_008626.2; NM_139241.3 |
| *FIG4* | FIG4 phosphoinositide 5-phosphatase | NG_007977.1; NM_014845.5 |
| *FLVCR1* | feline leukemia virus subgroup C cellular receptor 1 | NG_028131.1; NM_014053.3 |
| *GALC* | galactosylceramidase | NG_011853.2; NM_000153.3 |
| *GAN* | gigaxonin | NG_009007.1; NM_022041.3 |
| *GARS* | glycyl-tRNA synthetase | NG_007942.1; NM_002047.3 |
| *GDAP1* | ganglioside induced differentiation associated protein 1 | NG_008787.3; NM_018972.2 |
| *GJB1* | gap junction protein beta 1 | NG_008357.1; NM_000166.5 |
| *GJB3* | gap junction protein beta 3 | NG_008309.1; NM_024009.2 |
| *GNB4* | G protein subunit beta 4 | NG_033163.1; NM_021629.3 |
| *HADHB* | hydroxyacyl-CoA dehydrogenase/3-ketoacyl-CoA thiolase/enoyl-CoA hydratase (trifunctional protein), beta subunit | NG_007294.1; NM_000183.2 |
| *HARS* | histidyl-tRNA synthetase | NG_032158.1; NM_002109.5 |
| *HINT1* | histidine triad nucleotide binding protein 1 | NG_032998.1; NM_005340.6 |
| *HK1* | hexokinase 1 | NG_012077.1; NM_033498.2 |
| *HOXD10* | homeobox D10 | NG_008133.2; NM_002148.3 |
| *HNRNPA1* | heterogeneous nuclear ribonucleoprotein A1 | NG_033830.1; NM_002136.3 |
| *HSPB1* | heat shock protein family B (small) member 1 | NG_008995.1; NM_001540.3 |
| *HSPB3* | heat shock protein family B (small) member 3 | NG_027758.1; NM_006308.2 |
| *HSPB8* | heat shock protein family B (small) member 8 | NG_007953.2; NM_014365.2 |
| *IGHMBP2* | immunoglobulin mu binding protein 2 | NG_007976.1; NM_002180.2 |
| *IKBKAP* | inhibitor of kappa light polypeptide gene enhancer in B-cells, kinase complex-associated protein | NG_008788.1; NM_003640.4 |
| *INF2* | inverted formin, FH2 and WH2 domain containing | NG_027684.1; NM_022489.3 |
| *IFRD1* | interferon related developmental regulator 1 | NG_027799.1; NM_001007245.2 |
| *KARS* | lysyl-tRNA synthetase | NG_028025.1; NM_001130089.1 |
| *KCC3/SLC12A6* | solute carrier family 12 member 6 | NG_007951.1; NM_133647.1 |
| *KIF1A/ATSV* | kinesin family member 1A | NG_029724.1; NM_001244008.1 |
| *KIF1B* | kinesin family member 1B | NG_008069.1; NM_015074.3 |
| *KIF5A* | kinesin family member 5A | NG_008155.1; NM_004984.2 |
| *LITAF* | lipopolysaccharide induced TNF factor | NG_009008.1; NM_004862.3 |
| *LMNA* | lamin A/C | NG_008692.2; NM_170707.3 |
| *LRSAM1* | leucine rich repeat and sterile alpha motif containing 1 | NG_032008.1; NM_138361.5 |
| *MARS* | methionyl-tRNA synthetase | NG_034077.1; NM_004990.3 |
| *MED25* | mediator complex subunit 25 | NG_017091.1; NM_030973.3 |
| *MEN2B/RET* | ret proto-oncogene | NG_007489.1; NM_020975.4 |
| *MFN2* | mitofusin 2 | NG_007945.1; NM_014874.3 |
| *MORC2* | MORC family CW-type zinc finger 2 | NG_046752.1; NM_001303256.2 |
| *MPZ* | myelin protein zero | NG_008055.1; NM_000530.7 |
| *MTMR2* | myotubularin related protein 2 | NG_008333.1; NM_016156.5 |
| *NAGLU* | N-acetyl-alpha-glucosaminidase | NG_011552.1; NM_000263.3 |
| *NDRG1* | N-myc downstream regulated 1 | NG_007943.1; NM_006096.3 |
| *NEFL* | neurofilament, light polypeptide | NG_008492.1; NM_006158.4 |
| *NGFB* | nerve growth factor | NG_007944.1; NM_002506.2 |
| *NTRK1* | neurotrophic receptor tyrosine kinase 1 | NG_007493.1; NM_002529.3 |
| *PDK3* | pyruvate dehydrogenase kinase 3 | NG_016762.1; NM_001142386.2 |
| *PHYH* | phytanoyl-CoA 2-hydroxylase | NG_012862.1; NM_006214.3 |
| *PLA2G6* | phospholipase A2 group VI | NG_007094.2; NM_003560.2 |
| *PLEKHG5* | pleckstrin homology and RhoGEF domain containing G5 | NG_007978.1; NM_020631.4 |
| *PMM2* | phosphomannomutase 2 | NG_009209.1; NM_000303.2 |
| *PMP22* | peripheral myelin protein 22 | NG_007949.1; NM_000304.3 |
| *POLG* | DNA polymerase gamma, catalytic subunit | NG_008218.2; NM_002693.2 |
| *PRDM12* | PR/SET domain 12 | NC_000009.12; NM_021619.2 |
| *PRNP* | prion protein | NG_009087.1; NM_000311.3 |
| *PRPS1* | phosphoribosyl pyrophosphate synthetase 1p | NG_008407.1; NM_002764.3 |
| *PRX* | periaxin | NG_007979.1; NM_181882.2 |
| *RAB7* | RAB7A, member RAS oncogene family | NG_008070.1; NM_004637.5 |
| *RARS* | arginyl-tRNA synthetase | NG_041809.1; NM_002887.3 |
| *REEP1* | receptor accessory protein 1 | NG_013037.1; NM_022912.2 |
| *RFVT2/SLC52A2* | solute carrier family 52 member 2 | NG_032872.1; NM_024531.4 |
| *RNF170* | ring finger protein 170 | NG_032868.1; NM_001160223.1 |
| *RYR1* | ryanodine receptor 1 | NG_008866.1; NM_000540.2 |
| *SBF1* | SET binding factor 1 | NG_041810.1; NM_002972.3 |
| *SBF2* | SET binding factor 2 | NG_008074.1; NM_030962.3 |
| *SCN11A* | sodium voltage-gated channel alpha subunit 11 | NG_033859.1; NM_014139.2 |
| *SCN9A* | sodium voltage-gated channel alpha subunit 9 | NG_012798.1; NM_002977.3 |
| *SETX* | senataxin | NG_007946.1; NM_015046.5 |
| *SH3TC2* | SH3 domain and tetratricopeptide repeats 2 | NG_007947.2; NM_024577.3 |
| *SLC25A19* | solute carrier family 25 member 19 | NG_008274.1; NM_001126121.1 |
| *SLC5A7* | solute carrier family 5 member 7 | NG_042267.1; NM_021815.4 |
| *SOX10* | SRY-box 10 | NG_007948.1; NM_006941.3 |
| *SPTLC1* | serine palmitoyltransferase long chain base subunit 1 | NG_007950.1; NM_006415.3 |
| *SPTLC2* | serine palmitoyltransferase long chain base subunit 2 | NG_028282.1; NM_004863.3 |
| *SPTLC3* | serine palmitoyltransferase long chain base subunit 3 | NC_000020.11; NM_018327.2 |
| *SURF1* | SURF1, cytochrome c oxidase assembly factor | NG_008477.1; NM_003172.3 |
| *TDP1* | tyrosyl-DNA phosphodiesterase 1 | NG_009164.1; NM_018319.3 |
| *TFG* | TRK-fused gene | NG_027821.1; NM_006070.5 |
| *TRIM2* | tripartite motif containing 2 | NG_041788.1; NM_015271.4 |
| *TRPV4* | transient receptor potential cation channel subfamily V member 4 | NG_017090.1; NM_021625.4 |
| *VCP* | valosin containing protein | NG_007887.1; NM_007126.3 |
| *VRK1* | vaccinia related kinase 1 | NG_016293.1; NM_003384.2 |
| *WNK1* | WNK lysine deficient protein kinase 1 | NG_007984.3; NM_213655.4 |
| *YARS* | tyrosyl-tRNA synthetase | NG_008408.1; NM_003680.3 |
